# Pediatric airway compression in aortic arch malformations: a multidisciplinary approach

**DOI:** 10.3389/fped.2023.1227819

**Published:** 2023-07-21

**Authors:** Francesca Petreschi, Antonella Coretti, Federica Porcaro, Alessandra Toscano, Cosimo Marco Campanale, Marilena Trozzi, Aurelio Secinaro, Annalisa Allegorico, Renato Cutrera, Adriano Carotti

**Affiliations:** ^1^ Pediatric Pulmonology and Cystic Fibrosis Unit, Bambino Gesù Children’s Hospital, IRCCS, Rome, Italy; ^2^ Perinatal Cardiology Unit, Bambino Gesù Children’s Hospital, IRCCS, Rome, Italy; ^3^ Airway Surgery Unit, Bambino Gesù Children’s Hospital, IRCCS, Rome, Italy; ^4^ Advanced Cardiothoracic Imaging Unit, Bambino Gesù Children’s Hospital, IRCCS, Rome, Italy; ^5^ Unit of Complex Cardiac Surgery with Innovative Techniques, Bambino Gesù Children’s Hospital, IRCCS, Rome, Italy

**Keywords:** aortic arch malformations, airway compression, stridor, tracheomalacia, surgical treatment

## Abstract

**Background:**

Aortic arch malformations (AAMs) should be suspected in the presence of persistent respiratory symptoms despite medical treatment or feeding problems at the pediatric age.

**Aim:**

We report a descriptive cohort of patients with AAMs and the local management protocol applied.

**Methods:**

A total of 59 patients with AAM were retrospectively reviewed. Three groups were identified: double aortic arch (DAA), group 1; complete vascular ring (non-DAA), group 2; and anomalous origin of the innominate artery (IA), group 3.

**Results:**

Prenatal diagnosis was available for 62.7% of the patients. In all, 49.2% of children were symptomatic. There was a significantly different prevalence of respiratory symptoms within the three groups: 73.7% in group 1, 24.2% in group 2, and 100% in group 3 (*p*-value: <0.001). Surgery was considered in the presence of symptoms in patients with DAA and in those with reduction of the tracheal section area greater than 50%. A total of 52.5% of the patients underwent surgical repair (median age 6 months). The median follow-up interval was 21.9 months. Respiratory symptoms improved in most symptomatic patients.

**Conclusions:**

No specific protocols are available for the management of patients with AAMs. Conservative treatment seems to be reasonable for asymptomatic patients or those with airway stenosis less than 50%. A close follow-up is necessary to identify early patients who become symptomatic.

## 1. Introduction

Symptoms of tracheoesophageal compression in children are generally associated with malformations of the aortic arch (AAMs). These malformations are represented by complete vascular rings (VRs), such as the double aortic arch (DAA) and right aortic arch (RAA) with the aberrant left subclavian artery (ALSA), or non-ring anomalies of the epiaortic vessels, such as the aberrant right subclavian artery and anomalous origin of the innominate artery (IA) from the aortic arch. The most common respiratory symptoms of airway compression are wheezing and stridor, followed by recurrent respiratory infections, barking cough, and poor exercise tolerance (1–3). On the other hand, dysphagia and failure to thrive are clinical manifestations of esophageal compression, which is mostly associated with complete VRs.

The true prevalence of AAMs is unknown as not all exhibit symptoms. However, VRs are estimated to account for approximately 1% of congenital cardiovascular anomalies (4, 5). Prenatal diagnosis using two-dimensional echocardiography (2D-ECHO) has certainly increased the frequency of AAMs being diagnosed either before or after birth when previously undiagnosed or discovered accidentally or after clinical manifestations. Postnatal diagnosis of AAMs, in addition to 2D-ECHO, includes multidetector computed tomography (MDCT) with angiography, cardiac magnetic resonance imaging (MRI), and airway endoscopy (AE), and the diagnostic approach depends on the predominant symptom and/or prenatal detection (3).

The purpose of this study is to report our experience in managing airway compression in patients with AAMs. We describe the symptoms, instrumental findings, treatment, and follow-up of a group of children affected by either complete VRs or anomalous origin of the IA. Additionally, we illustrate the diagnostic and therapeutic algorithm adopted in our tertiary center.

## 2. Methods

The clinical and instrumental data of all patients with AAMs enrolled for a multidisciplinary follow-up program at the Pediatric Pulmonology and Cystic Fibrosis Unit of Bambino Gesù Children's Hospital between January 1, 2016, and December 31, 2020, were retrospectively reviewed and analyzed.

Information regarding gender, gestational age, birth weight, type of AAM, presenting symptoms, associated anomalies, diagnostic investigations (2D-ECHO, MDCT with angiography, AE), need and indication for surgical treatment, and details of performed surgical procedures were obtained from electronic medical records with approval from the institutional review board.

The AAMs were classified into three groups based on the Stewart classification (1964) (6), the revised Stewart classification (2019) (7), and Backer's Congenital Heart Surgery Nomenclature and Database Project (8), as follows:

– *group 1:* DAA;
– *group 2:* complete VR non-DAA;
– *group 3:* anomalous origin of the IA (origin of brachiocephalic artery positioned too far to the left side of the aortic arch).

Combined airway endoscopy and MDCT with angiography were performed to assess the degree of airway lumen reduction. Flexible airway endoscopy under light sedation with inhaled sevoflurane to induce a conscious sedation was initially carried out in patients with stridor as the initial symptom and repeated when persistent symptoms were reported during the postoperative follow-up period. Airway endoscopy was also performed in asymptomatic patients when MDCT with angiography showed a degree of airway lumen reduction greater than 50% to obtain additional information.

MDCT with angiography was performed during the inspiration and expiration phase without general anesthesia to better define the anatomical relationship between the airway and vascular structures.

Tracheomalacia and bronchomalacia were defined as dynamic airway collapse detected during flexible bronchoscopy made under light sedation and spontaneous quiet breathing (9) or during MDCT with angiography in the end-expiration phase (10).

Renal and abdominal ultrasound, 2D-ECHO, and genetic testing (karyotype and CGH array) were considered to screen the presence of major associated abnormalities.

When surgery was indicated, the preferred surgical approach was the left posterolateral thoracotomy.

All patients underwent close medical follow-up, which included a pulmonology visit every 6 months up to 2 years of age, every 12 months up to 6 years of age, and every 24 months over 6 years of age. The patients also had multidisciplinary follow-up visits with a cardiologist and ENT surgeon every year up to the age of 6 years and every 2 years thereafter. Pulse oximetry or polysomnography and pulmonary function testing were performed based on reported symptoms and age.

The study design was approved by the local Committee Board, and written informed consent was obtained from the parents of all the children.

## 3. Statistical analysis

Statistical analysis was performed using STATA 13.1 (Lakeway Dr, TX, USA). Continuous variables were expressed as mean and standard deviation or as median and (interquartile) range, depending on the normality of the data distribution assessed by the Shapiro–Wilk test. Categorical variables were expressed as numbers and frequencies. Continuous variables were compared using the Kruskal–Wallis test and categorical variables using Fisher's exact test. A *p* < 0.05 was considered statistically significant.

## 4. Results

### 4.1. Types of aortic arch malformations

A total of 59 patients with complete VR (DAA and non-DAA) and anomalous origin of the IA were enrolled in the study, of whom 40 (67.8%) were male. The mean gestational age was 38.2 ± 2.2 weeks, and the mean birth weight was 3,131.6 ± 527.1 grams.

Three anatomic variants of AAMs were identified, as above mentioned.

**Figure 1:**
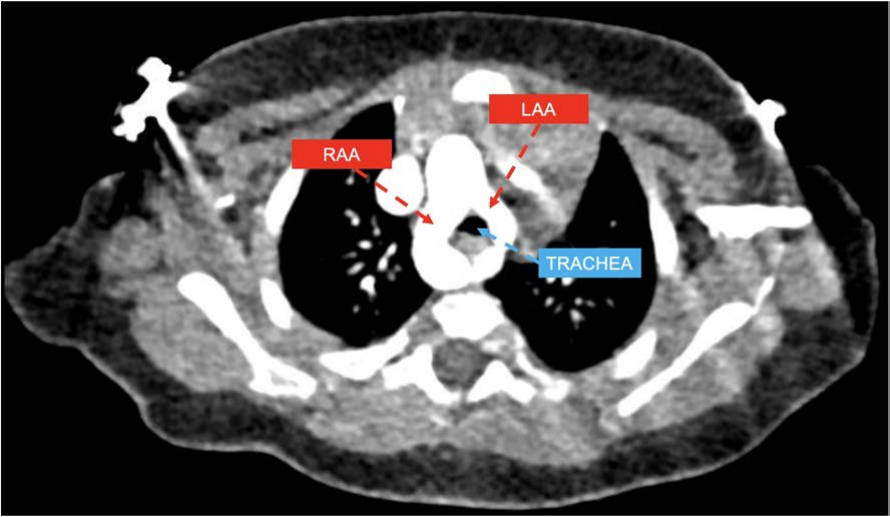
CT axial view showing double aortic arch with dominant right aortic arch (RAA) and hypoplastic left aortic arch (LLA) compressing the trachea.

In all, 19 patients with DAA (example provided in Figure 1) were classified in *group 1*. Among them, 16 patients (84.2%) had right-sided arch dominance, 2 patients (10.5%) left sided dominance, and 1 patient (5.3%) had a balanced dominance.

**Figure 2:**
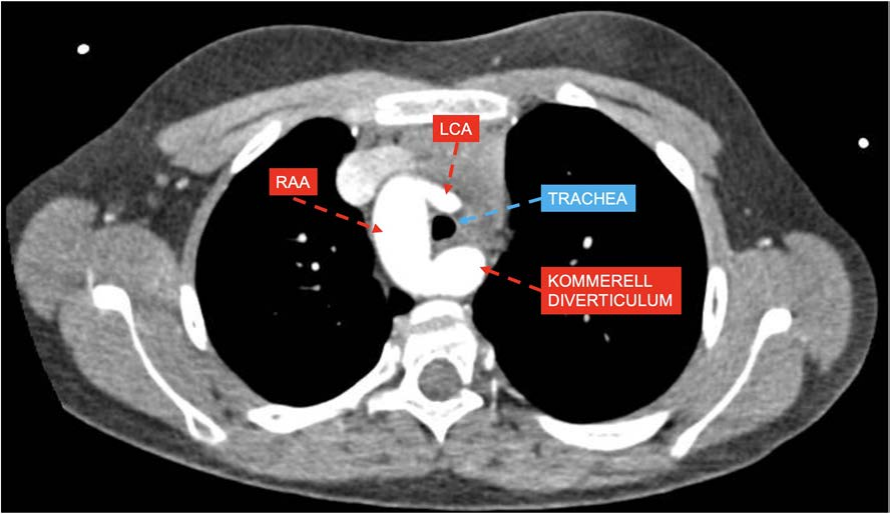
CT axial view showing right aortic arch (RAA) with aberrant left subclavian artery and Kommerell's diverticulum compressing the trachea with estimated lumen reduction of 55%; left coronary artery (LCA).

*Group 2* included 33 patients, 30 of whom had RAA with ALSA from a Kommerell's diverticulum of variable size (example provided in Figure 2), and the remaining 3 patients had RAA with mirror-image branching and left posterior ligamentum arteriosum.

**Figure 3:**
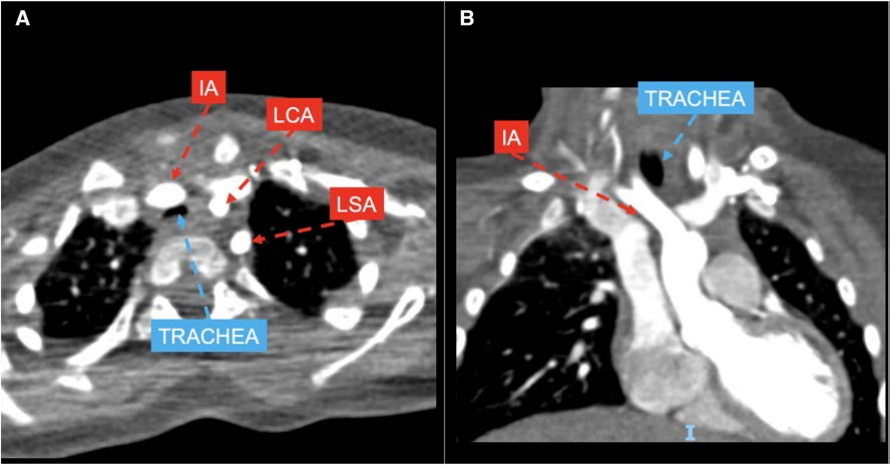
CT axial (**A**) and coronal (**B**) views showing left aortic arch with normal branching pattern and innominate artery (IA) anteriorly and laterally compressing the trachea; LCA, left coronary artery; LSA, left subclavian artery.

*Group 3* included 7 patients with anomalous origin of IA (example provided in Figure 3), in one case associated with bovine morphology (14.3%) and in another case with RAA (14.3%), leading to a variable degree of airway compression.

RAA with ALSA was the most common vascular abnormality (50.8%), followed by DAA (32.2%).

Associated malformations were found in 22 patients (37.3%), among whom 10 patients (16.19%) had atrial and/or ventricular septal defects and 5 patients (8.5%) had non-cardiac anomalies (including angiomas and renal malformations). A total of 7 patients (11.9%) received diagnosis of associated genetic mutations (microdeletion of chromosome 22 in 2 and MTHFR, SPG 15, and SRPX2 genes anomalies in the remaining 5 patients).

All this information is displayed in Table 1.

**Table 1:** Characteristics of the 59 patients enrolled in our study.

Variable	Patients *n* = 59
Male, *n* (%)	40 (67.8%)
Mean gestational age, weeks	38.2 ± 2.2
Type of vascular ring:	
DAA	19 (32.2%)
Arch dominance right sided	16 (84.2%)
Arch dominance left sided	2 (10.5%)
Balanced arches	1 (5.3%)
RAA	33 (55.9%)
ALSA + KD	30 (90.9%)
Mirror-image branching + left posterior LA	3 (9.1%)
Anomalous origin of the innominate artery	7 (11.9%)
LAA	6 (85.7%)
RAA	1 (14.3%)
Associated anomalies	22 (37.3%)
Cardiac defects (atrial and/or ventricular septal defects)	10 (16.9%)
Non-cardiac anomalies (angiomas and renal malformations)	5 (8.5%)
Genetic abnormalities (microdeletion of chromosome 22; MTHFR; SPG 15; SRPX2 anomalies)	7 (11.9%)

DAA, double aortic arch; RAA, right aortic arch; ALSA + KD, aberrant left subclavian artery + Kommerell's Diverticulum; LA; ligamentum arteriosus; LAA, left aortic arch.

### 4.2. Diagnostic tools

Prenatal diagnosis was performed on 37/59 patients (62.7%): 10 in *group 1*, 26 in *group 2*, and 1 in *group 3*. Among those diagnosed prenatally, 13 (35.1%) had symptoms at the first clinic visit after birth.

Postnatal diagnosis was accidental in 6/22 patients (27.3%) during evaluations conducted for other reasons, while it was based on clinical symptoms in 16/22 patients (72.7%).

For patients presenting with stridor as the predominant symptom, the initial investigation was flexible airway endoscopy, which was performed in 22/59 (37.3%) of cases. 2D-ECHO, MDCT with angiography, and cardiac MRI were performed in 61%, 96.6%, and 1.7% of cases, respectively.

Among the patients, 40 (67.8%) showed a maximum reduction in tracheal section area of less than 50%, while 19 (32.2) had a reduction greater than 50%. In addition, 25 patients (42.4%) were identified with tracheomalacia, and 8 of them had associated bronchomalacia. Data by group are shown in Table 2. The median age at the time of final diagnosis was 11.6 months (IQR: 3.5–38.5).

**Table 2:** Variable degree of airway lumen reduction detected with dynamic computed tomography and angiography and its distribution within the three groups.

	Total *n* = 59	*Group 1 n* = 19	*Group 2 n* = 33	*Group 3 n* = 7
Airway stenosis ≤50%, *n* (%)	40 (67.8%)	7 (36.8%)	27 (81.8%)	6 (85.7%)
Airway stenosis ≥50%, *n* (%)	19 (32.2%)	12 (63.2%)	6 (18.2%)	1 (14.3%)
Tracheomalacia, *n* (%)	25 (42.4%)	11 (57.9%)	10 (30.3%)	4 (57.1%)
Bronchomalacia, *n* (%)	8 (13.6%)	4 (21.1%)	3 (9.1%)	1 (14.3%)

Group 1, DAA; group 2, complete vascular rings non-DAA; group 3, anomalous origin of the innominate artery.

### 4.3. Clinical manifestations

Respiratory symptoms were found in 29 patients (49.2%), with 4 of them (6.8%) experiencing associated digestive symptoms. The most common respiratory symptom was stridor (14/59, 23.7%), followed by wheezing (9/59, 15.3%). Other respiratory symptoms included acute respiratory failure (3.4%), barking cough (3.4%), and combined recurrent upper airway infections, shortness of breath, and history of respiratory failure at birth with respiratory arrest and apnea (1.7%) (Table 3).

**Table 3:** Symptoms distribution among the three groups.

	Total *n* = 59	*Group 1 n* = 19	*Group 2 n* = 33	*Group 3 n* = 7
Stridor, *n* (%)	14 (23.7%)	5 (26.3%)	4 (12.1%)	5 (71.4%)
Wheezing, *n* (%)	9 (15.3%)	5 (26.3%)	3 (9.1%)	1 (14.3%)
Acute respiratory failure, *n* (%)	2 (3.4%)	2 (10.5%)	–	–
Barking cough, *n* (%)	2 (3.4%)	1 (5.3%)	–	1 (14.3%)
Recurrent UAI, *n* (%)	1 (1.7%)	1 (5.3%)	–	–
Shortness of breath, *n* (%)	1 (1.7%)	–	1 (3%)	–
Respiratory failure at birth, *n* (%)	1 (1.7%)	–	–	1 (14.3%)
Respiratory arrest, *n* (%)	1 (1.7%)	1 (5.3%)	–	–
Apnea, *n* (%)	1 (1.7%)	–	1 (3%)	–

UAI, upper airway infections.

One patient may have multiple symptoms.

Digestive symptoms consisted of dysphagia (3/59, 5.1%) and gastroesophageal reflux (GER) (1/59, 1.7%). Dysphagia occurred in 1 patient with RAA and ALSA, in 1 patient with anomalous origin of the IA, and in 1 child with DAA. GER was described in 1 patient with anomalous origin of the IA associated with RAA.

There was a significantly different prevalence of respiratory symptoms among the three groups: 73.7% in *group 1*, 24.2% in *group 2*, and 100% in *group 3* (*p* < 0.001). Among the common respiratory symptoms, only stridor showed a significant difference in prevalence (*p* = 0.005) when comparing the three groups (26.3% in *group 1*, 12.1% in *group 2*, and 71.4% in *group 3*). Stridor was significantly more evident in *group 3* than in *group 1* (*p* = 0.048).

The median age at symptoms onset was 2 months (IQR: 1–6), and no significant difference in age at symptoms onset was observed among the three groups. There was a significantly higher prevalence of respiratory symptoms at onset in *group 1* compared to *group 2* (*p* = 0.001).

### 4.4. Surgical treatments

Overall, 31 patients (52.5%) underwent surgical repair, with 16 of them having received a prenatal diagnosis: all children in *group 1*; 8 children in *group 2* (6 patients affected by RAA and ALSA, 2 patients by RAA, mirror-image branching, and left posterior LA); and 4 children in *group 3*.

The indications for surgery were as follows: (1) presence of persistent symptoms with impact on the child's quality of life, regardless of the degree of airway compression, (2) diagnosis of DAA, and (3) a reduction in the tracheal section area greater than 50%. The median age at the time of surgery was 6 months (IQR: 2.9–124), with no significant difference in the age at the time of surgery among the three groups. A total of 23 patients underwent surgical repair due to symptomatic presentations.

Meanwhile, 8 of the 30 asymptomatic patients (26.7%) affected by RAA with mirror-image branching and left posterior ligamentum arteriosum (*n* = 1) and RAA with ALSA (*n* = 2) had undergone surgery because of detection of maximum reduction of the tracheal section area greater than 50%; the remaining 5 patients had a diagnosis of DAA.

**Figure 4:**
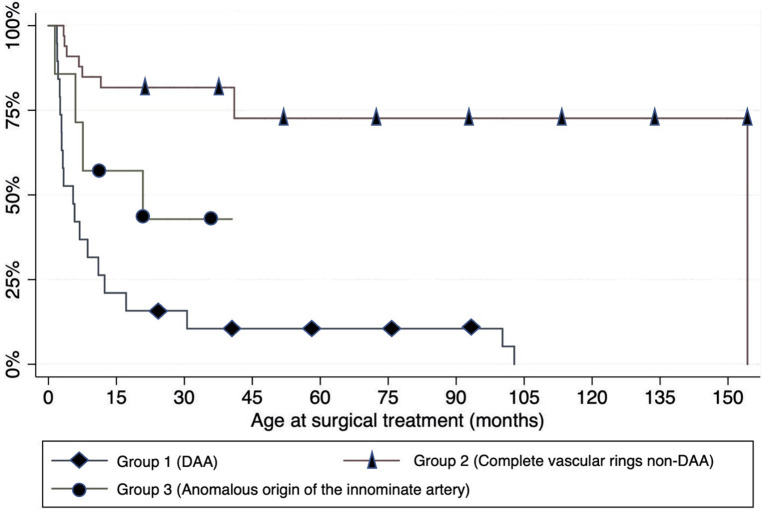
Timing and need of surgery (expressed in months) in 31 children: Kaplan–Meier analysis.

Most patients in each group underwent surgery before the age of 15 months. The need for surgery was significantly less frequent among the patients in group 2 (*p* < 0.001), as shown in Figure 4. A left posterolateral thoracotomy through the fourth intercostal space was the preferred surgical approach in the majority of cases (83.9%). Conversely, a sternotomy was performed only in case of anomalous origin of the IA.

All patients with DAA were surgically treated with transection of both the non-dominant AA and the LA. In 6 children with RAA and ALSA, some of whom had pre-operative dysphagia and stenosis of the origin of the ALSA and/or persistent intraoperative airway compression after LA transection, KD resection and ALSA reimplantation were carried out to treat symptoms and remove any potential substrate to avoid the risk of recurrence. Patients with RAA, mirror-image branching, and retroesophageal left ligamentum arteriosum underwent IA reimplantation, along with ligamentum arteriosum transection and aortopexy in one case and isolated ligamentum arteriosum transection in another. IA reimplantation was the treatment performed in 4 patients with severe airway compression due to anomalous IA, in 1 case associated to bovine morphology.

No surgery-related mortality was reported in our study population. Postoperative complications occurred in 10 patients, comprising four cases of pneumothorax, three cases of subcutaneous emphysema, two cases of combined pneumothorax and subcutaneous pneumothorax, and one case of chylothorax. All complications were resolved spontaneously. They were more prevalent among patients in *group 1* (*p =* 0.049), while no complications were recorded for those in *group 3*.

The median interval between diagnosis and surgery was 3.18 months (IQR: 2.03–4.75) for 23 symptomatic patients, while the median interval between the symptoms onset and surgery was 4.44 months (IQR: 1.66–9.39).

### 4.5. Follow-up visits

All the enrolled patients started the pulmonary outpatient follow-up program. The median interval between surgery and the initial check-up visit was 4.39 months (IQR: 2.16–11.31), and between the first and last follow-up visit it was 21.9 months (IQR: 13.8–28.8). Two patients were lost to follow-up: one from the group of operated patients and one from the group of non-operated patients.

In total, 57 patients (96.6%) completed the follow-up, of whom 30 were surgically treated. At the last follow-up visit, 95.6% of patients (*n *= 22) who underwent surgery because symptomatic and reported complete resolution of symptoms. However, one patient from *group 1* required a second surgical procedure (aortopexy) to recover from symptoms. Only two patients with DAA, surgically treated at 9 and 2 months of age, experienced shortness of breath at the end of the observation period.

Among the eight asymptomatic patients who underwent surgery, seven (87.5%) remained asymptomatic throughout the follow-up period, while one patient (12.5%) developed stridor even at rest and required re-intervention. However, all eight children were asymptomatic at the last follow-up visit.

Among the 28 patients who did not receive surgical treatment, 22 (78.6%, 17 with a prenatal diagnosis) remained asymptomatic from the beginning to the end of the follow-up period, while 5 patients (17.9%) with mild symptoms at the time of diagnosis (4 with a prenatal diagnosis) had a progressive improvement in their clinical condition. Overall, all 27 children were free of symptoms by the end of the observation period.

## 5. Discussion

A shared and uniform program for the management of patients with AAMs and respiratory complaints that involves pediatricians, cardiologists, pulmonologists, and both ENT and cardiothoracic surgeons is still to be implemented (1, 2, 4, 5). In light of this, we aimed to report the findings of our study that was performed in a small group of patients diagnosed with AAM, followed-up and treated, when required, in a single institution over the last five years.

Grouping our patients, we distinguished DAA as a separate category due to its tendency to cause greater and progressive compression of the trachea and esophagus, leading to earlier clinical manifestations compared to other vascular causes of airway compression (11–13). Our analysis showed that RAA with ALSA was the most common type of VR, as recently confirmed by other studies (14, 15), often associated with a deletion of 22q11.2 (16, 17).

In agreement with other series (4, 18–23), respiratory symptoms were predominant in our series, including mainly stridor and wheezing. We observed a significantly higher prevalence of respiratory symptoms at presentation in *group 1* compared to *group 2* as DAA exerts stronger compression on the airways and digestive tract compared to other complete vascular rings.

In our cohort, the median age at the onset of symptoms was 2 months. However, no significant difference in the age of symptoms onset among the three groups was detected, contrary to findings reported by other authors (21).

The median age of patients in our series diagnosed after birth aligned with previous reports (23–26). A different issue concerns prenatal diagnostics (21, 25), which supported us in cases of DAA, where proactive treatment was initiated even in asymptomatic patients to prevent the long-term effects of prolonged tracheal compression (27). On the other hand, for cases of RAA with ALSA, fetal diagnosis supported us in targeted patient monitoring, anticipating postnatal diagnostics, and allowing for the early detection of symptoms that might have otherwise gone unnoticed. Finally, with reference to the anomalous origin of IA, prenatal diagnostics had no role as this condition presents symptoms only after birth.

MDCT imaging supported by angiography was the primary diagnostic examination at our center for postnatal diagnosis. Indeed, it is able to accurately delineate the anatomy of AAMs and associated tracheal or bronchial pathology in a short acquisition time (28) during free breathing and without general anesthesia. Dynamic MDCT has also proven to be a diagnostic non-invasive exam for trachea-bronchomalacia (29). Consequently, children with suspected AAM underwent MDCT with angiography either at the onset of symptoms or routinely 6 months after birth in the case of prenatal diagnosis.

The combination of imaging and airways endoscopic evaluation allowed us to detect tracheomalacia and bronchomalacia in 42.4% and 13.6% of patients, respectively. This agrees with what has already been reported by Yubbu and colleagues, who described complete vascular rings as the principal cardiovascular cause of tracheomalacia (53.6%) and bronchomalacia (10.7%) (30).

Surgery remains the only effective treatment for symptomatic AAMs (19). In our entire cohort, the median age at surgery was 6 months, earlier than reported in other studies (13, 21, 31), probably due to the impact of prenatal diagnosis, particularly in asymptomatic patients. The surgical approach predominantly includes thoracotomy, as previously described (13, 32, 33), There was no intra- and postoperative mortality but only a limited number of minor complications, proving the safety of surgery in the case of AAMs (18, 21).

Our study has several limitations. Firstly, it had a retrospective design without a control group. Secondly, the small number of patients in *group 3* may explain the low strength of our statistical analysis. Finally, the short follow-up period prevented us from defining clear indications for surgery that predict the resolution of symptoms. Intuitively, we speculate that significant airway compression (>50% reduction in airway lumen) and/or the presence of symptoms may serve as significant predictive factors. Despite these speculations, we acknowledge the relatively weak strength of our conclusions.

On the other hand, the strength of our study lies in the exclusive inclusion of patients with AAMs related to vascular compression and the exclusion of those with associated tracheo-bronchial and pulmonary malformations. This approach minimized confounding factors that could influence the onset of respiratory symptoms.

**Figure 5:**
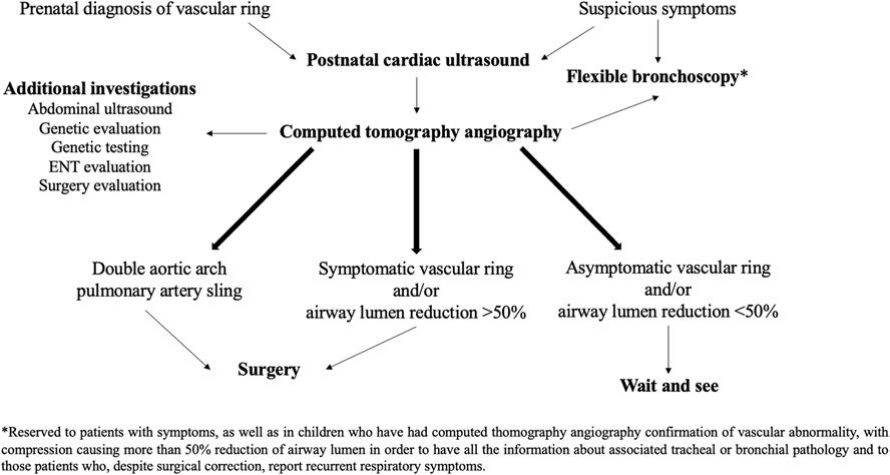
Diagnostic and therapeutic approach to pediatric airway compression in aortic arch malformations adopted at Bambino Gesù Children's Hospital.

In summary, our study describes our comprehensive approach to patients with AAMs, as shown in Figure 5. This approach involves: (1) the early diagnosis of vascular abnormalities; (2) timely surgical intervention in the case of DAA and/or respiratory or digestive symptoms impacting on the child's quality of life and/or airway stenosis exceeding 50%; and (3) close monitoring of asymptomatic patients and/or cases where vascular compression leads to less than 50% reduction in airway lumen (3). However, further prospective studies with long-term follow-up, ideally including a control group, are needed to establish clear guidelines for the diagnosis, treatment, and follow-up of patients affected by AAMs.

## Data Availability

The raw data supporting the conclusions of this article will be made available by the authors, without undue reservation.
